# Primary Vascular Dysregulation in Normal-Tension Glaucoma: Pathophysiology and Management

**DOI:** 10.7759/cureus.110693

**Published:** 2026-06-11

**Authors:** Arijita Banerjee, Pradosh Kumar Sarangi

**Affiliations:** 1 Physiology, Indian Institute of Technology Kharagpur, Kharagpur, IND; 2 Radiodiagnosis, All India Institute of Medical Sciences Deoghar, Deoghar, IND

**Keywords:** flammer syndrome, laser speckle contrast imaging, nocturnal hypotension, normal tension glaucoma, ocular autoregulation, ocular perfusion pressure, optical coherence tomography angiography, optic nerve head blood flow, primary vascular dysregulation

## Abstract

Normal-tension glaucoma (NTG) is a progressive optic neuropathy occurring in the absence of elevated intraocular pressure (IOP), implying that mechanisms beyond mechanical stress drive axonal loss. Primary vascular dysregulation (PVD), a constitutional impairment of vascular tone regulation independent of structural disease, is increasingly recognized as a central pathogenetic contributor to NTG. This review examines the evidence linking PVD to NTG, explores underlying mechanisms, and evaluates current and emerging management strategies targeting vascular dysregulation. A comprehensive narrative review of peer-reviewed literature was conducted using PubMed, MEDLINE, EMBASE, and Cochrane databases from 1985 to March 2026. Search terms included: normal-tension glaucoma, primary vascular dysregulation, Flammer syndrome, ocular blood flow, endothelin-1, calcium channel blockers, optic nerve head ischemia, autoregulation, and neurovascular coupling. Studies including randomized controlled trials, prospective observational cohorts, case-control studies, systematic reviews, and seminal basic science investigations were selected for inclusion based on relevance and quality. PVD encompasses impaired autoregulation of optic nerve head (ONH) perfusion, elevated endothelin-1 (ET-1) levels with relative nitric oxide (NO) deficiency, defective neurovascular coupling, mitochondrial dysfunction, oxidative stress, exaggerated nocturnal hypotension, and autonomic nervous system imbalance. Clinically, PVD-associated NTG is characterized by optic disc haemorrhages, focal notch-type cupping, central and paracentral visual field defects, and progressive loss despite controlled IOP. Pharmacological strategies targeting vascular dysregulation, including calcium channel blockers (CCBs), magnesium supplementation, *Ginkgo biloba* extract (GBE), and endothelin receptor antagonists, have demonstrated clinical benefit in selected patient cohorts. Optimization of nocturnal systemic blood pressure, lifestyle modifications, and stress reduction are integral non-pharmacological components of management. PVD represents a clinically actionable pathophysiological entity in a significant proportion of NTG patients. Recognition of the PVD phenotype should prompt a multimodal management approach that integrates IOP reduction with strategies targeting ocular perfusion and vascular stability. Future well-powered randomized controlled trials in phenotypically defined PVD-NTG populations are needed to establish definitive therapeutic guidelines and to validate emerging neuroprotective strategies.

## Introduction and background

Glaucoma represents the leading cause of irreversible blindness worldwide, affecting an estimated 80 million individuals globally, with projections suggesting this figure will reach 111 million by 2040. Among its heterogeneous subtypes, normal-tension glaucoma (NTG) presents a distinct and particularly challenging clinical entity. By definition, NTG is characterized by progressive glaucomatous optic neuropathy, manifested by optic disc cupping, retinal nerve fibre layer (RNFL) thinning, and corresponding visual field defects, occurring despite intraocular pressure (IOP) consistently within the statistically normal range of ≤21 mmHg [1,2].

This distinction has important clinical implications. It implies that the long-standing paradigm equating glaucoma pathogenesis primarily with elevated IOP is insufficient to explain the mechanism of optic nerve damage in NTG. While IOP reduction remains efficacious even within the normal range, as demonstrated by the landmark Collaborative Normal Tension Glaucoma Study (CNTGS), which showed a 35% reduction in progression risk with a 30% IOP reduction, a substantial proportion of NTG patients continue to progress despite well-controlled IOP. This residual progression necessitates consideration of alternative, IOP-independent pathogenetic mechanisms [3,4].

The prevalence of NTG varies significantly across ethnic populations. In East Asian populations, particularly Japanese, Korean, and Chinese, NTG accounts for 70%-90% of all primary open-angle glaucoma (POAG), while it represents approximately 30%-40% of POAG in Western populations. In Indian population, NTG constitutes an increasing proportion of open-angle glaucoma, with estimates ranging from 20% to 40% across different geographic cohorts. Over the past three decades, growing evidence has converged on vascular mechanisms as critical contributors to NTG pathogenesis. Foremost among these is the concept of primary vascular dysregulation (PVD), a constitutional impairment of vascular tone regulation independent of structural vascular disease, which renders the microvasculature of the optic nerve head (ONH) particularly susceptible to ischaemic insults. First systematically characterized by the Zurich group under Professor Josef Flammer, PVD, in its systemic clinical expression termed Flammer syndrome, encompasses a complex phenotype of vascular, metabolic, and neurological manifestations that collectively predispose affected individuals to optic nerve damage even in the absence of elevated IOP [5-7].

This narrative review aims to provide a comprehensive synthesis of current knowledge regarding the pathophysiological mechanisms linking PVD to NTG, to characterize the clinical manifestations and diagnostic tools relevant to this association, and to critically evaluate the therapeutic strategies, both established and emerging, that target vascular dysregulation in NTG. The ultimate goal is to promote a pathophysiology-informed, individualized approach to NTG management that extends beyond IOP reduction.

## Review

Methodology

A comprehensive narrative review of peer-reviewed literature was conducted using PubMed/MEDLINE, EMBASE, and the Cochrane Central Register of Controlled Trials (CENTRAL), covering publications from January 1985 to March 2026. The lower date boundary of 1985 was chosen to capture the earliest published clinical investigations into vascular mechanisms in low-tension glaucoma predating the formal characterisation of PVD. Search terms included: normal-tension glaucoma, primary vascular dysregulation, Flammer syndrome, ocular blood flow, endothelin-1, nitric oxide, autoregulation, neurovascular coupling, calcium channel blockers, nocturnal hypotension, ocular perfusion pressure, colour Doppler imaging, and optical coherence tomography angiography, applied individually and in Boolean combination (Appendix 1). Reference lists of retrieved articles were hand-searched to identify additional relevant sources. Over 600 records were initially identified across all databases; after title and abstract screening, approximately 220 were retained for full-text review, of which 78 met the inclusion criteria and were incorporated into this review. Of these, 46 are directly cited in the manuscript, with the remainder contributing to background contextualisation. We organized findings thematically by pathophysiological domain and by therapeutic class. Eligible studies included randomized controlled trials, prospective and retrospective cohort studies, case-control studies, systematic reviews, and seminal basic science investigations in adult human populations with confirmed NTG or primary vascular dysregulation. Studies restricted to animal models, paediatric populations, or secondary glaucoma without NTG-specific data were excluded. Publications were selected based on methodological quality, relevance to the review objectives, and contribution to mechanistic or therapeutic understanding. Given the heterogeneous study designs represented in the reviewed literature, a single validated quality appraisal tool was not uniformly applicable across all included studies; we aimed to balance methodological rigour with clinical relevance in our selection. This narrative review does not involve the collection of primary data, the enrolment of human participants, or the use of identifiable patient information. Accordingly, formal ethics committee approval was not required.

Primary vascular dysregulation: definition and systemic phenotype

Primary vascular dysregulation (PVD) is defined as a constitutional impairment of vascular tone regulation that occurs independently of structural vascular pathology such as atherosclerosis, vasculitis, or embolic disease. It is distinguished from secondary vascular dysregulation, which arises as a consequence of systemic conditions such as diabetes mellitus, systemic hypertension, connective tissue disorders, or thrombophilia, by its intrinsic, likely genetically determined nature. In its systemic clinical expression, PVD has been conceptualized as Flammer syndrome. Individuals with Flammer syndrome characteristically display a slender body habitus with low body mass index (BMI, often <22 kg/m²), low systemic blood pressure (including orthostatic hypotension), cold extremities (particularly hands and feet), heightened sensitivity to medications and environmental stimuli, altered pain perception, increased stress reactivity, perfectionist personality traits, sleep disturbances, increased dream intensity, and, in some cases, tinnitus and enhanced sensitivity to low oxygen environments [8-10].

At the vascular level, PVD is characterized by an exaggerated and inappropriately sustained vasoconstrictive response to physiological stimuli, particularly cold exposure, emotional stress, and hypoxia. This vasospastic tendency reflects a fundamental imbalance in the regulation of vasoactive mediators, with a predominance of vasoconstrictive forces, particularly endothelin-1 (ET-1), relative to vasodilatory mediators, principally nitric oxide (NO). The result is recurrent, episodic vasoconstriction in the microvasculature, with potential consequences for end-organ perfusion in sites such as the ONH, choroid, and retrobulbar vessels [11].

PVD appears to have a genetic substrate, although specific causative genes have not been comprehensively identified. A family history of similar constitutional features is frequently elicited in affected individuals. Interestingly, PVD is more prevalent in females than in males, a finding that may relate to hormonal modulation of vascular tone. The prevalence of PVD in the general population is estimated at approximately 20%-30%, although this figure depends heavily on the diagnostic criteria applied. From an ophthalmological perspective, PVD is not limited to NTG. It has been implicated in the pathogenesis of central serous chorioretinopathy (CSC), optic disc, retinal vein occlusion, and non-arteritic anterior ischaemic optic neuropathy (NAION). However, it is in the setting of NTG that the PVD vascular axis has been most systematically investigated and where its therapeutic relevance is most clearly established [12,13].

Pathophysiological mechanisms linking PVD to NTG

Impaired Optic Nerve Head Autoregulation

The optic nerve head (ONH) is one of the most metabolically demanding regions of the central nervous system per unit volume and is critically dependent on adequate and constant blood supply. Under physiological conditions, the ONH vasculature exhibits robust autoregulatory capacity, the ability to maintain stable perfusion despite fluctuations in systemic blood pressure and IOP, within a range of mean arterial pressures approximately between 50 and 150 mmHg. This autoregulation is mediated by myogenic responses of vascular smooth muscle, metabolic sensing, and endothelial-derived vasoactive factors. In PVD, associated NTG, this autoregulatory capacity is fundamentally impaired. Studies employing laser Doppler flowmetry (LDF), scanning laser Doppler flowmetry, and laser speckle contrast imaging (LSCI) have consistently demonstrated that NTG patients show reduced baseline ONH blood flow and a diminished or absent autoregulatory response to changes in perfusion pressure. A seminal contribution from Konieczka and colleagues showed that retinal blood flow in NTG patients fails to increase appropriately in response to flickering light stimulation, a paradigm for neurovascular coupling (NVC), with some patients demonstrating a paradoxical vasoconstrictive response [14]. The clinical consequence of impaired autoregulation is that NTG patients are rendered vulnerable to episodic ONH ischaemia during physiological perturbations, particularly nocturnal blood pressure dipping, postural hypotension, and vasospastic episodes triggered by cold or stress. These recurrent ischaemic insults, individually subclinical, may cumulatively produce progressive retinal ganglion cell (RGC) apoptosis and axonal degeneration characteristic of glaucomatous neuropathy [14,15].

Endothelin-1 and Nitric Oxide Imbalance

Endothelin-1 (ET-1) is the most potent and sustained endogenous vasoconstrictor known. It is synthesized primarily by vascular endothelial cells and acts on two receptor subtypes: endothelin-A (ETA) receptors on vascular smooth muscle cells, which mediate vasoconstriction; and endothelin-B (ETB) receptors, which exist on both endothelial cells (mediating vasodilation via NO release) and smooth muscle cells (mediating further vasoconstriction). In PVD, ETA receptor-mediated vasoconstriction predominates. Elevated plasma and aqueous humour ET-1 concentrations have been consistently demonstrated in NTG patients compared to both healthy controls and patients with high-tension glaucoma. Beyond its haemodynamic effects, ET-1 exerts direct neurotoxic effects on RGCs through activation of ET receptors on ganglion cells, induction of mitochondrial dysfunction, and promotion of apoptotic cascades. Animal models in which ET-1 is administered to the optic nerve or intravitreally reproduce ONH cupping and RGC loss analogous to those observed in glaucoma [16].

Nitric oxide (NO), produced by endothelial NO synthase (eNOS) from L-arginine, is the primary physiological counterbalance to ET-1. NO promotes vascular smooth muscle relaxation, inhibits platelet aggregation, and possesses anti-inflammatory and antioxidant properties. In PVD-associated NTG, NO bioavailability is reduced, attributable to increased consumption by reactive oxygen species (ROS), eNOS uncoupling, and substrate insufficiency. Elevated plasma levels of asymmetric dimethylarginine (ADMA), an endogenous competitive inhibitor of eNOS, have been reported in a subset of NTG patients, providing a mechanistic explanation for NO deficiency. Elevated plasma homocysteine, which impairs endothelial function through oxidative and thrombotic mechanisms, is also overrepresented in NTG patients, particularly those with optic disc haemorrhages [17].

Neurovascular Coupling Deficits

Neurovascular coupling (NVC) refers to the physiological mechanism by which increased neuronal metabolic activity in the retina triggers proportionate local vasodilation to meet increased oxygen and glucose demands. This process depends on the coordinated function of the neurovascular unit, comprising neurons, Müller cells, astrocytes, pericytes, and endothelial cells, and involves signalling pathways including glutamate-mediated astrocytic calcium waves, prostaglandin release, epoxyeicosatrienoic acid (EET) production, and purinergic signalling. In NTG with PVD, NVC is profoundly impaired. The retinal vascular response to luminance flicker stimulation, quantified using the Dynamic Vessel Analyzer (DVA) (IMEDOS Systems UG, Jena, Germany) as the percentage increase in retinal vessel diameter, is markedly attenuated or paradoxically reversed in NTG patients. This reversed response, vasoconstrictive rather than vasodilatory in response to functional demand, is pathognomonic of severe NVC impairment and has not been observed in healthy individuals or high-tension glaucoma patients. It implies that during periods of retinal neuronal activity, the ONH and peripapillary microvasculature in NTG may be exposed to relative hypoperfusion rather than the compensatory hyperaemia that should occur [18].

Mitochondrial Dysfunction and Oxidative Stress

The unmyelinated axons of the ONH are uniquely dependent on mitochondrial oxidative phosphorylation for adenosine triphosphate (ATP) production, as they lack the energy efficient saltatory conduction of myelinated axons. This renders them exquisitely sensitive to disruptions in mitochondrial function and oxidative homeostasis. In PVD-associated NTG, transient ischaemia reperfusion cycles generate a burst of reactive oxygen species (ROS), particularly superoxide anion and hydrogen peroxide, that overwhelm endogenous antioxidant defences. Aqueous humour and serum markers of oxidative stress, including 8-isoprostane, malondialdehyde, 8-hydroxydeoxyguanosine, and protein carbonyls, are elevated in NTG patients relative to controls, confirming systemic and local oxidative burden. Mitochondrial DNA copy number, a surrogate marker of mitochondrial mass and bioenergetic reserve, is reduced in peripheral blood cells of some NTG patients, suggesting a systemic mitochondrial deficiency beyond the eye. The genetic architecture of NTG further implicates mitochondrial pathways. Optineurin (OPTN), one of the first genes identified as causative in familial NTG, encodes a protein involved in mitochondrial autophagy (mitophagy), Nuclear factor kappa-light-chain-enhancer of activated B cells (NF-κB) signalling, and Golgi maintenance. TANK-binding kinase 1 (TBK1) duplications, associated with NTG, encode a kinase critical for selective autophagy. These genetic insights suggest that impaired mitochondrial quality control, when superimposed on vascular insufficiency, creates a synergistically toxic environment for ONH axons [19].

Nocturnal Hypotension and Ocular Perfusion Pressure

Systemic blood pressure normally declines by 10%-20% during sleep, the so-called "dipping" phenomenon, which is a physiological adaptation mediated by the circadian regulation of the autonomic nervous system. Ocular perfusion pressure (OPP), calculated as mean arterial pressure minus IOP (OPP = 2/3 mean arterial pressure (MAP) - IOP), correspondingly declines during sleep. In healthy individuals with intact ONH autoregulation, this nocturnal OPP reduction is well-compensated. However, in NTG patients with impaired autoregulation and PVD, even a normal dipping pattern may result in critical ONH hypoperfusion. Multiple studies employing 24-hour ambulatory blood pressure monitoring (ABPM) have documented a significantly higher prevalence of extreme dipping (>20% nocturnal BP reduction) in NTG patients compared to age-matched controls and high-tension glaucoma patients. Inverse dipping, a paradoxical nocturnal rise in BP associated with autonomic dysfunction, may also occur and is linked to an increased risk of glaucomatous progression. The mean nocturnal OPP has been identified as an independent predictor of visual field progression rate in NTG cohort studies, underscoring the haemodynamic relevance of these nocturnal changes [20].

Autonomic Nervous System Dysregulation

The autonomic nervous system plays a key role in regulating vascular tone and haemodynamic responses to postural changes, stress, and circadian variation. In PVD, there is consistent evidence of autonomic imbalance characterized by relative sympathetic hyperactivity and reduced parasympathetic tone. This manifests clinically as orthostatic hypotension, reduced baroreflex sensitivity, and impaired heart rate variability (HRV). HRV, the variation in time intervals between successive heartbeats, is a validated non-invasive measure of autonomic function. Reduced time domain HRV indices (standard deviation of normal-to-normal R-R (SDNN), root mean square of successive differences between normal heartbeat intervals (RMSSD)) and altered frequency domain components have been reported in NTG patients, indicating sympathovagal imbalance. This impaired autonomic flexibility contributes to haemodynamic instability, reduces adaptive vasoregulatory capacity, and may amplify the vasoconstrictive effects of emotional stress and cold exposure in PVD individuals. Elevated plasma catecholamines, particularly norepinephrine, in PVD individuals further promote systemic and local vasoconstriction via alpha adrenergic receptor activation in the peripapillary vasculature. The adrenergic innervation of the ONH microvasculature, while sparse compared to other vascular beds, appears to play a modulatory role in normal ONH blood flow regulation, and its dysregulation in PVD may contribute to abnormal vascular responses [21,22].

Clinical features of PVD-associated NTG

The recognition of PVD as a distinct pathological substrate in NTG has important clinical implications, both for disease characterization and for therapeutic decision making. Several ophthalmological and systemic features, when present in combination, suggest a vascular aetiology for NTG in individual patients.

Systemic Phenotypic Markers

Patients with PVD-associated NTG frequently exhibit the systemic phenotype of Flammer syndrome: a slender body habitus with BMI <22 kg/m², low systemic blood pressure (often systolic <110 mmHg), cold extremities, a tendency to fainting or near fainting (particularly in crowded, hot, or emotionally stressful environments), sleep difficulties, and heightened drug sensitivity. Enquiry about Raynaud phenomenon, an exaggerated vasoconstrictive response of the digits to cold, is particularly informative, as this represents one of the most overt manifestations of PVD. Eliciting a family history of similar constitutional traits further supports the PVD phenotype [23]. A structured clinical probability framework for diagnosing Flammer syndrome/PVD, including the diagnostic features and supportive objective investigations, is provided in Appendix 2.

Optic Disc Morphology

Optic disc haemorrhages (Drance haemorrhages) are a hallmark of NTG and are significantly more frequent in NTG than in high-tension glaucoma (3- to 5-fold higher incidence). They represent localized ischaemic events at the disc margin, most commonly at the inferior or inferotemporal poles, and are pathognomonic of vascular pathology. Their presence on serial fundus photography should prompt evaluation for PVD and optimization of vascular risk factors. The pattern of optic nerve cupping in NTG tends to differ from that in high-tension glaucoma. Focal, notch-type excavation, particularly at the inferior or inferotemporal neuroretinal rim, is more characteristic of NTG and reflects the focal ischaemic mechanism of PVD. In contrast, concentric, symmetric cupping is more typical of high-tension glaucoma, where mechanical effects of elevated IOP act diffusely. The neuroretinal rim area in NTG tends to be relatively preserved overall, with localized notching rather than generalized thinning, at least in earlier stages [24].

Visual Field Characteristics

Visual field defects in NTG characteristically differ from those in high-tension glaucoma in their location, depth, and proximity to fixation. Defects in NTG tend to be deeper, more steeply sloped, and more centrally located, often within the central 10°, compared to the more peripheral Bjerrum scotomas typical of high-tension glaucoma. Paracentral scotomas threatening or encroaching on fixation are overrepresented in NTG and are of particular concern given their functional significance for reading and fine visual tasks. This central predilection for visual field loss in NTG has been attributed to the selective vulnerability of the papillomacular bundle, the axons serving the central retina, which traverse the temporal ONH and are supplied by short posterior ciliary artery branches that are particularly susceptible to vasospasm. The superior hemifield is more commonly affected than the inferior in NTG, a distribution opposite to that seen in high-tension glaucoma, further supporting a distinct pathogenetic mechanism. When serial OCT demonstrates progressive RNFL thinning or ganglion cell complex (GCC) loss in the absence of IOP elevation, or when visual field indices deteriorate at a clinically significant rate despite adequate IOP control, vascular re-evaluation is warranted. Ocular disc haemorrhages on routine fundus assessment in a patient with apparent IOP control should similarly prompt investigation of vascular risk [25].

Diagnostic assessment of vascular dysregulation in NTG

Ocular Blood Flow Imaging

Optical coherence tomography angiography (OCT-A) has emerged as the most clinically accessible tool for assessing the ONH and peripapillary microvasculature in NTG. It enables non-invasive, high-resolution mapping of the superficial and deep capillary plexuses, the radial peripapillary capillary (RPC) network, and the choroidal vasculature without the need for dye injection. Studies consistently demonstrate reduced RPC vessel density in the temporal peripapillary region in NTG compared to controls and to high-tension glaucoma, correlating with RNFL thickness and visual field mean deviation. Serial OCT-A may serve as a structural biomarker of vascular compromise and disease progression [26].

Colour Doppler imaging (CDI) of retrobulbar vessels provides functional haemodynamic data, peak systolic velocity (PSV), end-diastolic velocity (EDV), and resistivity index (RI), in the ophthalmic artery (OA), central retinal artery (CRA), and short posterior ciliary arteries (SPCAs). Elevated RI in the SPCAs and CRA, reflecting increased vascular resistance, is a consistent finding in NTG with vascular dysregulation. The SPCAs are of particular clinical relevance as they supply the laminar and prelaminar ONH, the site of primary axonal injury in glaucoma. Laser speckle contrast imaging (LSCI) enables wide-field, real-time mapping of relative ONH blood flow and is particularly suited to assessing the dynamic NVC response to flickering light. The magnitude and direction of blood flow change in response to flicker provides a functional index of NVC integrity. An attenuated or paradoxically negative response is characteristic of PVD-NTG [27].

Dynamic Vessel Analysis

The Dynamic Vessel Analyzer (DVA) enables quantification of retinal vessel diameter changes in response to standardized flickering light stimulation (16 Hz, 0.8 Hz, alternating luminance). In healthy individuals, a 3%-5% diameter increase in retinal arterioles occurs, reflecting intact NVC. In NTG patients with PVD, this response is attenuated or reversed. DVA thus provides a functional readout of retinal NVC and can identify patients with impaired vascular reactivity who may be candidates for vascular-targeted therapy [28].

Systemic Vascular Investigations

Twenty-four-hour ABPM is an essential investigation in all NTG patients, with particular attention to nocturnal dipping pattern, extreme dipping (>20%), non-dipping or inverse-dipping profiles, and the minimum nocturnal OPP. Calculation of mean nocturnal OPP requires concurrent IOP measurement (preferably with intraocular telemetric sensors or rebound tonometry at multiple time points). The identification of pathological nocturnal haemodynamics is directly actionable, particularly in patients on antihypertensive therapy [29].

Cold provocation testing, measuring digital arterial pressure or nailfold capillaroscopy before and during hand immersion in ice-cold water, provides objective evidence of PVD. An abnormal vasoconstrictive response (>20% reduction in finger blood pressure or blanching of nail bed capillaries) supports the PVD diagnosis. Nailfold capillaroscopy can also identify morphological microvascular abnormalities (e.g., megacapillaries, avascular areas, irregular loops) that indicate constitutional vascular fragility [30].

Biomarker measurement should include fasting plasma homocysteine, plasma ADMA (where available), serum magnesium, and fasting lipid and glucose profiles. Elevated homocysteine (>15 μmol/L) warrants B vitamin supplementation (folate, B12, B6), which has been shown to reduce homocysteine levels and may improve endothelial function. Plasma ET-1 measurement remains primarily a research tool but may be considered in specialized centres [31].

Management of primary vascular dysregulation in NTG

The management of PVD-associated NTG is inherently multimodal. IOP reduction remains the only intervention with established level I evidence for reducing NTG progression and should be maintained as the cornerstone of therapy. However, in patients who progress despite adequate IOP control, or in whom vascular risk factors are prominent, targeted vascular interventions are rationally and empirically justified. Table 1 summarizes the principal vascular-targeted management strategies in PVD-associated NTG, along with their mechanisms, levels of evidence, and key clinical considerations.

**Table 1:** Summary of Vascular-Targeted Management Strategies in PVD-Associated NTG SR: slow release; Mg: magnesium; PAF: platelet activating factor; ET-1: endothelin-1; ETA: endothelin A receptor; NO: nitric oxide; LBB: latanoprostene bunod; IOP: intraocular pressure; OPP: ocular perfusion pressure; ABPM: ambulatory blood pressure monitoring; PVD: primary vascular dysregulation; MBSR: mindfulness-based stress reduction; CDP: citicoline; NTG: normal-tension glaucoma, CBT: cognitive behavioural therapy; RCT: randomized controlled trial; EGb: *Ginkgo biloba* extract; TDS: three times daily; ETB: endothelin-B. Refs: [32-41]. This table was created by the authors (original).

Intervention	Mechanism	Evidence Level	Key Consideration
Calcium channel blockers (e.g., nifedipine SR 20 mg/d)	L-type Ca²⁺ channel blockade → vasodilation, ↓ vasospasm	Level II-III (RCTs and observational)	Monitor for hypotension; preferred in cold-hands phenotype
Magnesium supplementation (121.5 mg elemental Mg, twice daily)	Physiological Ca²⁺ antagonist, vascular smooth muscle relaxation	Level III (small RCTs)	Check serum Mg; well-tolerated with GI monitoring
*Ginkgo biloba* extract EGb 761 (40 mg TDS)	Antioxidant, anti-PAF, vasodilatory, rheological	Level III (RCTs)	Safe; check drug interactions (anticoagulants)
ET-1 receptor antagonists (e.g., bosentan, ambrisentan)	Block ETA/ETB → reverse ET-1-mediated vasoconstriction	Level IV (animal/pilot)	Not approved for glaucoma; experimental use only
NO-donating drugs (latanoprostene bunod, L-arginine)	↑ NO bioavailability → vasodilation + IOP reduction	Level II (LBB for IOP)	Dual mechanism; vascular benefit supplementary to IOP
Morning dosing of antihypertensives	Reduces nocturnal dipping → improves nocturnal OPP	Level II (RCT by Quaranta)	Requires 24-h ABPM to identify eligible patients
Aerobic exercise (≥150 min/week moderate)	Improves endothelial function, ↑ retinal blood flow, ↓ sympathetic tone	Level III (observational)	Broadly beneficial; individualize intensity
Dietary modification (Mediterranean diet, ↑ nitrate-rich foods)	↑ dietary NO precursors; antioxidant, anti-inflammatory	Level IV (expert consensus)	Recommend leafy greens, omega-3 fatty acids
Cold avoidance and thermal protection	Reduces vasospastic triggers in PVD	Level V (clinical principle)	Behavioural; simple to implement
Stress reduction (MBSR, CBT, relaxation)	↓ sympathetic activation → ↓ vasospastic episodes, ↓ ET-1	Level IV (extrapolated from other conditions)	Particularly useful in high-stress personality phenotype
Citicoline (CDP-choline, 500-1,000 mg/d oral)	Neuroprotection, membrane phospholipid synthesis, cholinergic modulation	Level III (small RCTs)	Supplement to primary management; ongoing trials
B-vitamin supplementation (folate, B12, B6)	Reduces homocysteine → improves endothelial function	Level III	Indicated if plasma homocysteine >15 μmol/L

Calcium Channel Blockers

Calcium channel blockers (CCBs), particularly dihydropyridine class agents (nifedipine, amlodipine, felodipine) and the phenylalkylamine verapamil, are the most studied pharmacological agents for targeting vascular dysregulation in NTG. Their principal mechanism involves blockade of voltage-gated-type calcium channels in vascular smooth muscle, reducing intracellular calcium concentration and thereby promoting smooth muscle relaxation and vasodilation. In PVD, where pathological vasospasm is mediated partly through calcium-dependent mechanisms, CCBs address the proximate mechanism of vasoconstriction [32].

Netland et al. conducted an early randomized, double-masked crossover study demonstrating that oral nifedipine significantly increased retinal blood flow as measured by scanning laser Doppler flowmetry and improved visual field indices in NTG patients [33]. Notably, an observational analysis from the CNTGS found that NTG patients concurrently taking systemic CCBs were approximately 50% less likely to progress, independent of IOP, than non-users. CCBs must be used judiciously. Their systemic hypotensive effect, while generally modest at low doses of slow-release preparations, can exacerbate orthostatic hypotension in patients with constitutional low blood pressure (a feature of PVD/Flammer syndrome), potentially worsening rather than improving ONH perfusion. Careful blood pressure monitoring before and after initiation is essential. Slow-release nifedipine at the lowest effective dose (20 mg daily) is preferred over immediate-release formulations. Topical ophthalmic CCBs remain experimental [4,33].

Magnesium Supplementation

Magnesium (Mg²⁺) acts as a natural calcium channel antagonist, competing with calcium at smooth muscle voltage-gated channels and thereby promoting vasodilation. Magnesium deficiency is associated with increased vasomotor tone and endothelial dysfunction. Gaspar et al. published the seminal prospective study in which oral magnesium supplementation (121.5 mg elemental magnesium twice daily for one month) produced statistically significant improvement in peripheral visual field thresholds in NTG patients with Raynaud phenomenon, with a concurrent increase in retinal blood flow on LDF [34]. A follow-up study extended these findings over six months, demonstrating sustained benefit. Magnesium supplementation is attractive for its excellent safety profile, low cost, and physiological rationale. Gastrointestinal side effects (diarrhoea) are the principal adverse effects and can be mitigated by using magnesium glycinate or threonate formulations rather than magnesium oxide. The optimal dose and formulation for NTG management remain to be established by larger, longer-term trials. Serum magnesium levels do not reliably reflect intracellular or tissue magnesium status, complicating the identification of deficient patients; erythrocyte magnesium measurement may be a superior biomarker [34].

Ginkgo Biloba Extract

*Ginkgo biloba* extract (GBE), specifically the standardized *Ginkgo biloba* extract (EGb) 761 preparation, exerts multiple vascular and neuroprotective effects relevant to NTG: it inhibits platelet-activating factor (PAF), reduces blood viscosity through erythrocyte membrane stabilization, scavenges free radicals, increases retinal blood flow via NO-dependent vasodilation, and modulates ET-1 signalling. Quaranta et al. demonstrated in a prospective, randomized, placebo-controlled study that EGb 761 (40 mg three times daily for four weeks) produced statistically significant improvements in preexisting visual field mean deviation in NTG patients [35]. A subsequent longer-term observational study by the same group associated GBE use with slower rates of visual field progression over a mean follow-up exceeding three years [35].

GBE is generally well tolerated, but potential drug interactions must be considered, particularly with anticoagulant and antiplatelet agents, given its PAF inhibitory and antiplatelet properties. The evidence base, while encouraging, derives from relatively small studies with short follow-up durations. GBE should not be regarded as a substitute for proven IOP-lowering or vascular therapy but represents a rational, low-risk adjunctive option [36].

Endothelin Receptor Antagonists

The central role of ET-1 in PVD-associated NTG makes endothelin receptor antagonists (ERAs) a compelling therapeutic target. Bosentan, a dual ETA/ETB receptor antagonist approved for pulmonary arterial hypertension, has been shown in experimental models to improve ONH perfusion and reduce RGC loss when ET-1 is administered to induce optic nerve ischaemia. Selective ETA receptor antagonists (atrasentan, sitaxentan, ambrisentan) theoretically offer a more favourable profile by preserving ETB-mediated endothelial vasodilation and NO release while blocking pathological vasoconstriction. Clinical application of ERAs in NTG is currently experimental. No ERA has received regulatory approval for glaucoma. Key barriers include systemic side effects (hepatotoxicity with bosentan, peripheral oedema, teratogenicity), the absence of adequately powered randomized clinical trials in NTG populations, and the lack of commercially available topical formulations. The development of topical selective ETA antagonists for ocular use is an active area of pharmaceutical research and represents a potentially transformative future direction [37,38].

Nitric Oxide-Enhancing Strategies

Strategies to restore or enhance NO bioavailability in PVD-associated NTG address the mechanistic counterpart to ET-1 excess. Latanoprostene bunod, a prostaglandin F2α-nitric oxide donor conjugate, releases NO upon esterase cleavage in the trabecular meshwork and ciliary body, providing both enhanced IOP reduction (superior to latanoprost in head to head trials) and local vasodilatory effects. While its primary regulatory indication is IOP reduction, the NO component may provide additional vascular benefit in NTG with PVD, although this has not been formally studied [39].

L-arginine, the eNOS substrate and precursor to NO, has been administered as an oral supplement in conditions of endothelial dysfunction, improving endothelium-dependent vasodilation in cardiovascular disease contexts. Its utility in NTG remains unexplored in dedicated trials. Phosphodiesterase type 5 (PDE-5) inhibitors, which prevent cyclic GMP degradation and thereby potentiate NO-mediated vasodilation, have been shown to acutely increase retinal and choroidal blood flow. However, concerns regarding transient IOP elevation and the possibility of precipitating non-arteritic anterior ischaemic optic neuropathy (NAION) in predisposed individuals currently preclude routine recommendation [40].

Optimization of Systemic Blood Pressure Management

Optimization of nocturnal blood pressure represents a key and often underutilized therapeutic lever in NTG management. In patients with systemic hypertension receiving antihypertensive pharmacotherapy, the timing of medication administration should be guided by 24-hour ABPM findings. Patients demonstrating extreme nocturnal dipping or in whom OPP falls below a critical threshold (mean OPP <50 mmHg is frequently cited as clinically significant) should have antihypertensive medication switched from evening to morning dosing wherever medically feasible [41].

In patients with constitutional hypotension, a hallmark of PVD/Flammer syndrome, the focus is on preventing symptomatic hypotension and haemodynamic instability. Practical measures include ensuring adequate salt and fluid intake (in the absence of cardiac or renal contraindications), wearing compression stockings to reduce venous pooling and orthostatic hypotension, rising slowly from the supine position, and avoiding large carbohydrate meals (which promote postprandial hypotension). Where pharmacological intervention is required, fludrocortisone (a mineralocorticoid) or midodrine (an alpha-adrenergic agonist) may be considered in consultation with a general physician or cardiologist [42].

Lifestyle Modifications

Regular moderate-intensity aerobic exercise exerts systemic benefits directly relevant to PVD and NTG. Exercise improves endothelial function through upregulation of eNOS activity and increased shear stress, reduces blood viscosity through favourable effects on haematocrit and fibrinogen, modulates autonomic tone toward parasympathetic predominance, and has been shown to increase retinal blood flow and vascular reactivity in multiple studies. Walking, swimming, and cycling at moderate intensity for ≥150 minutes per week are appropriate recommendations for most NTG patients, tailored to comorbidities and individual tolerance [43].

Smoking cessation is mandatory in all NTG patients. Tobacco smoking promotes endothelial dysfunction, increases oxidative stress, elevates plasma fibrinogen and blood viscosity, and induces sympathomimetic vasospasm through nicotinic receptor activation. Even passive (secondhand) smoke exposure has measurable detrimental effects on retinal microvascular function. Clinicians should proactively counsel patients and facilitate cessation with pharmacotherapy (varenicline, nicotine replacement) as needed. Dietary patterns enriched in nitrate-containing vegetables (beetroot, spinach, arugula), omega-3 polyunsaturated fatty acids (oily fish, walnuts, flaxseed), and polyphenol-rich foods (berries, dark chocolate, green tea) support endothelial NO production, reduce inflammation, and improve microvascular function. The Mediterranean dietary pattern encompasses these elements and is associated with reduced cardiovascular event rates and improved endothelial markers in multiple large-scale prospective studies, providing a sound evidence base for its recommendation in NTG patients with PVD [44].

Stress Reduction and Psychological Interventions

Psychological stress is a well-documented trigger of vasospasm in PVD, mediated through activation of the sympathoadrenal axis, increased circulating catecholamines and ET-1, and increased platelet reactivity. Patients with Flammer syndrome often exhibit perfectionist, high-achieving personality profiles with heightened emotional reactivity, traits associated with chronic sympathetic hyperactivation. Targeted psychological interventions may therefore modulate the vascular phenotype in PVD [45].

Mindfulness-based stress reduction (MBSR), an eight-week structured programme incorporating meditation, body scan, and yoga, has demonstrated reductions in plasma cortisol, improvements in HRV, and enhanced endothelium-dependent vasodilation in hypertensive and cardiovascular disease populations. Cognitive behavioural therapy (CBT) addresses maladaptive cognitive patterns that sustain stress responses. Biofeedback, particularly HRV biofeedback training, directly targets autonomic balance and has shown promise in conditions of dysautonomia. These approaches, while not yet tested in formal NTG clinical trials, represent low-risk patient-centred adjuncts that align with the pathophysiology of PVD [46].

Limitations of current evidence

Despite the compelling mechanistic framework linking PVD to NTG, the evidence base for vascular-targeted therapy has several limitations that constrain the formulation of definitive clinical guidelines. First, most intervention studies have been small (n<100), short-term (<1 year), and lack adequate randomization and masking. Second, phenotypic heterogeneity within NTG, with some cases driven primarily by mechanical factors and others primarily by vascular mechanisms, makes it difficult to identify the subgroup most likely to benefit from vascular therapy without validated biomarkers. Third, surrogate endpoints (retinal blood flow, vessel density on OCT-A, plasma ET-1) have not been validated as predictors of long-term visual field outcomes. Fourth, reporting bias and publication of positive studies inflate perceived effect sizes. Fifth, the lack of topical formulations for many vascular agents forces reliance on systemic drugs with dose-limiting side effects. Future research priorities include large, multicentre, randomized controlled trials of CCBs and ERAs in phenotypically characterized PVD-NTG patients; validation of OCT vessel density and DVA flicker response as surrogate progression markers; development and clinical testing of topical ETA antagonists; genome-wide association studies of vascular NTG; and precision medicine trials stratifying patients by genotype and vascular biomarker profile.

## Conclusions

PVD is a central, actionable driver of NTG through multiple synergistic mechanisms, including impaired ONH autoregulation, ET-1/NO imbalance, defective neurovascular coupling, mitochondrial dysfunction, and autonomic dysregulation that cumulatively cause IOP-independent RGC loss. Identifying the PVD phenotype via ABPM, OCT-A, CDI, DVA flicker testing, and biomarker profiling justifies a multimodal approach combining IOP reduction with vascular-targeted therapy, of which slow-release nifedipine carries the strongest evidence, supported by magnesium, *Ginkgo biloba*, and lifestyle modification.

Looking ahead, wider availability of OCT-A, laser speckle imaging, and circadian telemetry will sharpen individual vascular risk profiling, while emerging agents, such as topical ETA antagonists, mitochondria-targeted antioxidants, and NO-donating drops, promise more precise intervention. The immediate clinical priority is integrating vascular phenotyping into routine NTG assessment; the research priority is large, well-powered RCTs in phenotypically defined PVD-NTG populations.

## Appendices

Appendix 1

Search Strategy

The following databases were searched for this narrative review: PubMed/MEDLINE, EMBASE, and Cochrane Central Register of Controlled Trials (CENTRAL). The search was conducted from January 1985 to March 2026. The search strategy employed the following terms in various combinations using Boolean operators:

normal tension glaucoma [MeSH/Title/Abstract] OR low tension glaucoma [MeSH/Title/Abstract] OR primary vascular dysregulation [MeSH/Title/Abstract] OR Flammer syndrome [MeSH/Title/Abstract] OR ocular blood flow [MeSH/Title/Abstract] OR ocular perfusion pressure [MeSH/Title/Abstract] OR optic nerve head blood flow [MeSH/Title/Abstract] OR endothelin,1 [MeSH/Title/Abstract] OR nitric oxide [MeSH/Title/Abstract] OR autoregulation [MeSH/Title/Abstract] OR neurovascular coupling [MeSH/Title/Abstract] OR calcium channel blockers [MeSH/Title/Abstract] OR nifedipine glaucoma [MeSH/Title/Abstract] OR magnesium glaucoma [MeSH/Title/Abstract] OR Ginkgo biloba glaucoma [MeSH/Title/Abstract] OR endothelin receptor antagonist glaucoma [MeSH/Title/Abstract] OR nocturnal hypotension glaucoma [MeSH/Title/Abstract] OR retinal vessel density OCT angiography glaucoma [MeSH/Title/Abstract] OR colour Doppler imaging glaucoma [MeSH/Title/Abstract] OR dynamic vessel analysis glaucoma

Inclusion criteria: peer-reviewed original research articles (randomized controlled trials, prospective and retrospective cohort studies, case-control studies), systematic reviews, meta-analyses, and authoritative narrative reviews published in English. Articles were excluded if they addressed only animal models without discussion of human relevance, involved populations exclusively under 18 years of age, or were published only as conference abstracts.

Appendix 2

Diagnostic Criteria for Flammer Syndrome/PVD

No universally validated diagnostic criteria for Flammer syndrome exist. However, a clinical probability framework proposed by Konieczka et al. (2014) [9] includes the following features, with the presence of ≥4 considered supportive of the diagnosis:

1. Slender body habitus (BMI <22 kg/m² in adults)

2. Low systemic blood pressure (systolic <110 mmHg or habitual orthostatic symptoms)

3. Cold extremities (subjective report of persistently cold hands and/or feet)

4. Raynaud phenomenon (episodic digital colour change in response to cold/stress)

5. Increased sensitivity to medications (particularly analgesics, anaesthetics, and cardiovascular drugs)

6. Sleep disturbances with increased dream recall

7. Perfectionist, high-achieving personality profile

8. Objective evidence of impaired vascular reactivity (DVA flicker response, cold provocation test, nailfold capillaroscopy)

Objective confirmation may be sought through abnormal DVA flicker response (attenuated or reversed); abnormal cold provocation test (>20% reduction in digital arterial pressure); nailfold capillaroscopy abnormalities; elevated plasma ET-1; reduced plasma NO metabolites; elevated ADMA; and abnormal 24-hour ABPM (extreme dipping pattern).
